# Important factors for effective patient safety governance auditing: a questionnaire survey

**DOI:** 10.1186/s12913-018-3577-9

**Published:** 2018-10-20

**Authors:** Saskia C. van Gelderen, Marieke Zegers, Paul B. Robben, Wilma Boeijen, Gert P. Westert, Hub C. Wollersheim

**Affiliations:** 1 Radboud university medical center, Radboud Institute for Health Sciences, IQ healthcare, P.O. Box 9101, 6500 HB Nijmegen, the Netherlands; 2 Erasmus School of Health Policy & Management, Erasmus University Rotterdam, Rotterdam, the Netherlands; 3 Department of Quality and Safety, Radboud university medical center, Nijmegen, the Netherlands

**Keywords:** Audit, Clinical governance, Patient safety, Hospital, Quality improvement

## Abstract

**Background:**

Audits are increasingly used for patient safety governance purposes. However, there is little insight into the factors that hinder or stimulate effective governance based on auditing. The aim of this study is to quantify the factors that influence effective auditing for hospital boards and executives.

**Methods:**

A questionnaire of 32 factors was developed using influencing factors found in a qualitative study on effective auditing. Factors were divided into four categories. The questionnaire was sent to the board of directors, chief of medical staff, nursing officer, medical department head and director of the quality and safety department of 89 acute care hospitals in the Netherlands.

**Results:**

We approached 522 people, of whom 211 responded. Of the 32 factors in the questionnaire, 30 factors had an agreement percentage higher than 50%. Important factors per category were ‘audit as an improvement tool as well as a control tool’, ‘department is aware of audit purpose’, ‘quality of auditors’ and ‘learning culture at department’. We found 14 factors with a significant difference in agreement between stakeholders of at least 20%. Amongst these were ‘medical specialist on the audit team’, ‘soft signals in the audit report’, ‘patients as auditors’ and ‘post-audit support’.

**Conclusion:**

We found 30 factors for effective auditing, which we synthesised into eight recommendations to optimise audits. Hospitals can use these recommendations as a framework for audits that enable boards to become more in control of patient safety in their hospital.

**Electronic supplementary material:**

The online version of this article (10.1186/s12913-018-3577-9) contains supplementary material, which is available to authorized users.

## Background

Hospital boards and executives are responsible for ensuring that healthcare is delivered in a safe manner in their hospitals [1, 2]. Unsafe healthcare can lead to patient harm and unnecessary costs [3]. However, healthcare incidents continue, suggesting that hospital boards are still not in control when it comes to assuring patient safety [4–7]. Boards have different instruments at their disposal, but the ongoing discussion on how to be in control is nowhere near ending [8, 9].

**Fig. 1:**
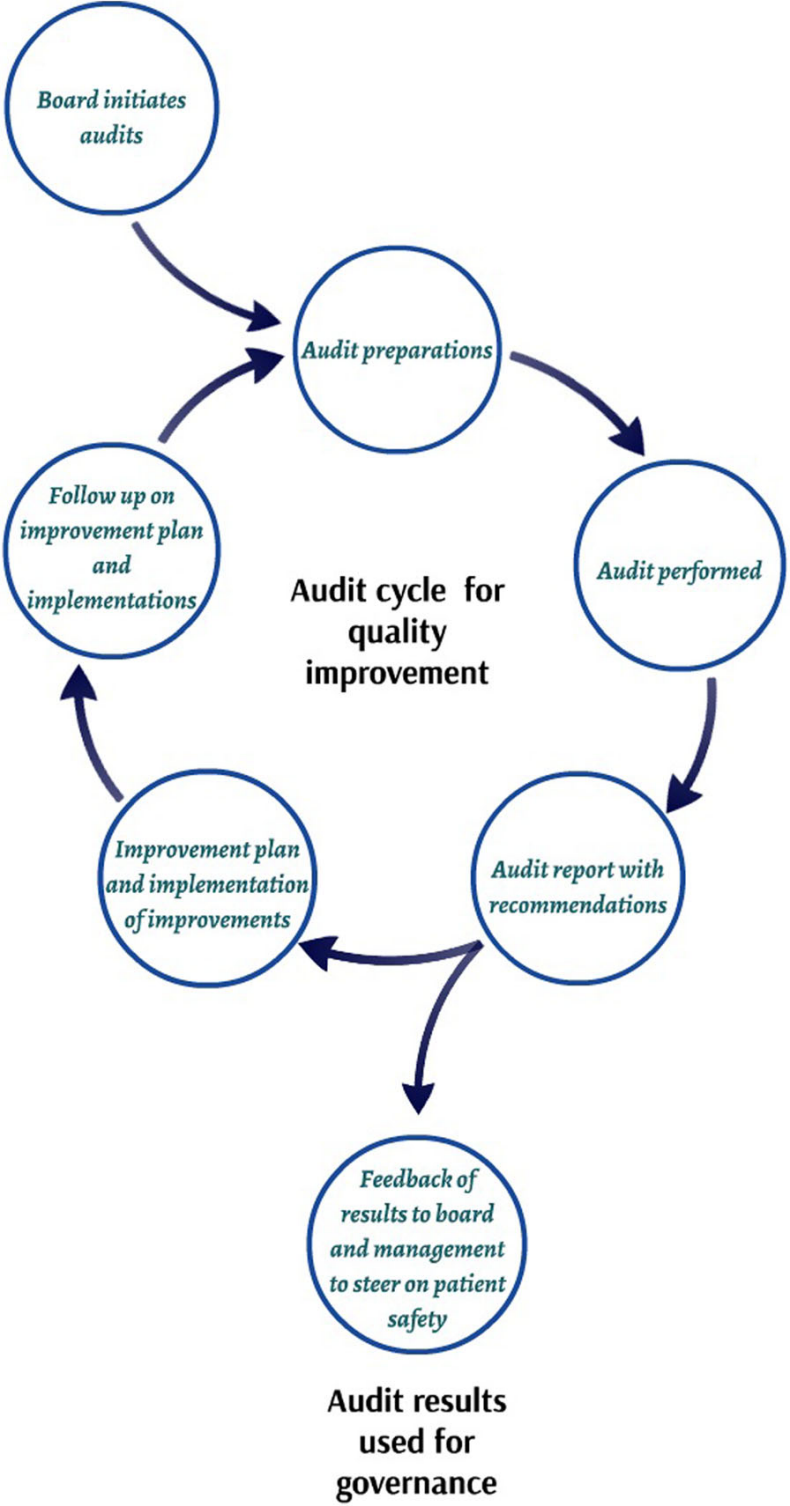
Audit cycle for quality improvement and governance purposes [13]

One of the instruments that hospital boards and executives can use for this purpose is auditing. Auditing is a multiple-source method that evaluates whether standards and regulations are being followed [10]. In contrast to clinical auditing, which focusses on one specific clinical area and is initiated by healthcare professionals [11, 12], the audit to which we refer in this study is a hospital-wide audit, which focusses on auditing all departments on a periodic basis and is initiated by hospital boards [13], as is shown in Fig. 1.

This hospital-wide audit focusses on patient safety and quality of care. It is not an audit focussed on one medical speciality, nor a financial audit. This type of auditing is based on a peer-to-peer approach; auditors are colleagues from a different department than the department being audited. The audit involves site visits, interviews, document analysis, surveys and observations.

Insight into the effects of auditing is the subject of previous studies, showing that auditing leads to small but potentially important improvements in professional behaviours and practice (e.g. appropriate prescribing of medicines) and experienced patient safety by patients [10–12, 14]. However, insight into the factors that hinder or stimulate effective use of auditing for boards and managers to govern patient safety is lacking. This might be a missing link while auditing is widely used for quality improvement purposes [15]. Earlier research that did focus on barriers to and facilitators for effective auditing is either from years ago [15], or focussed on clinical audits, without taking into account the different stakeholders in the governance and audit process but, for example, auditors only [16]. Studying a more complete overview of stakeholders, including auditors, clinicians and hospitals boards, provides a more complete overview of essential components for auditing. Recently, a qualitative study was carried out to study which factors hinder or stimulate hospital-wide audits for governance purposes perceived by a wide range of stakeholders [13]. Findings from this study indicate that four central themes influence effective auditing; two themes consisting of factors related to the audit itself (Organisation and content of audits, and Competences and composition of audit team) and two themes regarding contextual factors (Board positioning of audits, and Cultural factors and attitudes towards auditing).

Quantifying the importance of these factors might help hospital boards and executives determine which factors should be a priority when optimising audits and which factors need less attention. Therefore, the aim of this study is to quantify the factors that influence effective auditing for hospital boards and executives to govern patient safety.

## Methods

### Setting

In the Netherlands, hospitals are obliged by law to systematically monitor, control and improve the quality of care [17]. An audit is one instrument that is widely used for this purpose, as the existence of an audit system is a condition for accreditation. Hospitals are interested in accreditation because it gives third parties, e.g., healthcare consumers and healthcare insurers, the assurance of safe healthcare [18]. The average audit cycle in Dutch hospitals is 4 years; in this period, every department must have been audited [19]. The audit consists of several elements, including a document analysis of policy documents, interviews, surveys and observations. After an audit, the audit team writes an audit report which is communicated to the head of the audited department. These results are disseminated to the hospital board for governance purposes. In some hospitals, the audit results are discussed in planning and control cycle meetings [10].

### Study design

In this cross sectional study, a questionnaire in all acute care hospitals in the Netherlands (*n* = 89) was undertaken between November 2014 and January 2015. At each hospital, different stakeholders were asked to fill in a questionnaire. Stakeholders involved in governance and the auditing process were selected based on a qualitative study [13] in which a stakeholder analysis [20–23] was performed. The following six stakeholders per hospital received the questionnaire: (1) a member of the board of directors, (2) the chief of medical staff, (3) the nursing officer, (4) the head of the department of orthopaedic surgery, (5) the head of the internal medicine department and (6) the director of the quality and patient safety department (or unit). We wanted to combine a surgical and a medical department and therefore choose the departments of orthopaedic surgery and internal medicine. An electronic questionnaire was sent by email. The email included the purpose of the study and a statement that anonymous and confidential handling of data was ensured. When a respondent indicated that there was no audit in the hospital, the questionnaire was stopped at that hospital. A reminder was sent 2 weeks later. Informed consent was implied by completing and sending in the questionnaire.

### Questionnaire development

Barriers and facilitators incorporated into the questionnaire were based on outcomes of the previous qualitative study on barriers and facilitators for effective auditing, in which in-depth interviews with boards of directors (*n* = 5), boards of supervisors (n = 5), heads of medical departments (medical specialists and clinical managers) (*n* = 12) and quality managers and auditors (*n* = 21) were held. In-depth qualitative analysis of these interviews, using the Grounded Theory approach, resulted in 32 barriers and facilitators, from which four themes emerged [13]. These categories were (1) board positioning of audits, (2) organisation and content of audits, (3) competences and composition of audit team and (4) cultural factors and attitudes towards auditing [13]. The 32 barriers and facilitators were translated into neutral statements for the questionnaire. The questionnaire was piloted amongst one clinician who was also head of a medical department, three experts on auditing who were also auditors, and one expert on hospital governance. The pilot consisted of filling in the questionnaire to test its feasibility and discussing the questions with one of the researchers (SvG) after completion to test whether questions were understandable.

To determine the importance of factors influencing effective auditing, respondents were asked to rate on a 6-point Likert scale (1 = fully disagree, 6 = fully agree) the extent to which they agreed or disagreed with the 35 statements, including three statements regarding the participation of patients in auditing. The questionnaire further consisted of general questions, e.g., age, gender, function and type of hospital, and one open-ended question at the end of the questionnaire to give respondents the option to add comments. The questionnaire is added in Additional file 1.

### Analysis of the data

The questionnaire data were analysed using IBM SPSS Statistics version 20. The responses to the statements regarding factors that influence effective auditing were dichotomised per item as ‘disagree’ from 1 to 3 and ‘agree’ from 4 to 6. We performed logistic regressions for each question, to test whether there was a significant difference between subgroups in the percentage of agreement. Significance was set at *p* < 0.05. A higher agreement percentage for a factor meant that more participants perceived that factor as important [24, 25]. Factors were ranked from 1 (most important) to 32 (least important) based on this agreement percentage. When multiple factors had the same agreement percentage, we ranked them based on their item scores, including their 95% confidence interval (CI). We performed an exploratory factor analysis [26], to see whether unknown structures appeared from the data. We did not find these, so we included every factor in the ranking.

Three statements in the questionnaire were not directly related to audit effectiveness, but explored the reasons for not including patients in the audit team (‘patients are not suited to be an auditor because they are not objective’; ‘patients are not suited to be an auditor because they do not have sufficient knowledge regarding healthcare guidelines’; ‘patients are not suited to be an auditor because confidentiality cannot be secured’). Hence, they were not included in the ranking of important factors for effective auditing, but were included in the logistic regression to test whether there was difference between stakeholders regarding these questions.

We sent the questionnaire to six respondents per hospital, leading to a two level nested structure within the data set. Testing whether data cluster within hospitals arose was not possible because of the low number of observations per hospital (average observation of 2.6 per hospital), meaning that the chance of data clustering was small and multilevel analysis was not necessary.

## Results

### Response

**Fig. 2:**
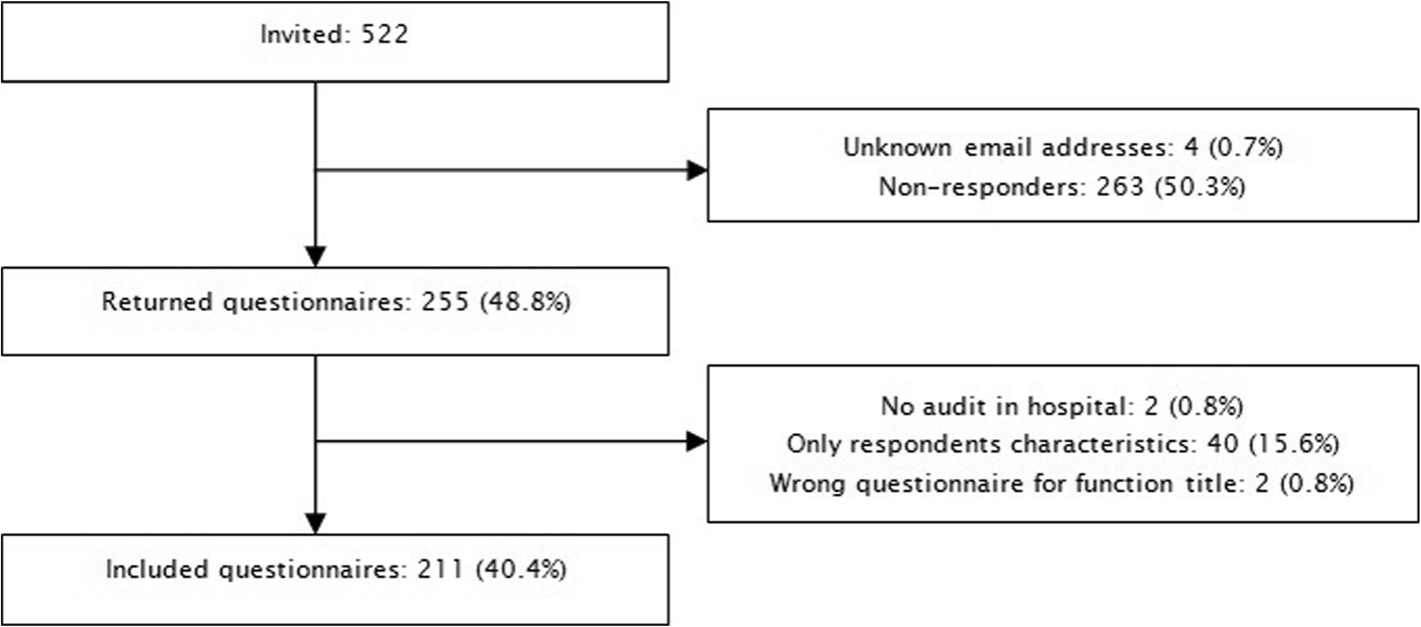
Flowchart of the inclusion process

Of the 89 Dutch hospitals, two did not have an audit system and six did not respond (response rate 91%). Of the 81 hospitals, the response rate per hospital type was 100% for university medical centres (*n* = 8), 96% for tertiary teaching hospitals (*n* = 27) and 86% for general hospitals (*n* = 46). Figure 2 shows the inclusion process.

We intended to send 534 questionnaires; however, not every function was present in every hospital and, therefore, 12 questionnaires could not be sent. Of the 522 questionnaires sent, 211 questionnaires were completed, resulting in an overall response rate of 40%. The response rate varied per stakeholder type: 25% for boards of directors (*n* = 22), 39% for chiefs of medical staff (*n* = 35), 50% for nursing officers (*n* = 40), 31% for heads of the two medical departments together (*n* = 55) and 69% for directors of the quality and patient safety departments (*n* = 59).

### Characteristics of respondents

Of the 211 respondents, 12% worked in an academic medical centre, 33% worked in a tertiary teaching hospital and 55% worked in a general hospital. Regarding functions, 10% were boards of directors, 17% were chief of medical staff, 19% were nursing officers, 26% were heads of medical departments, i.e., medical specialists and clinical managers, and 28% were directors of quality and patient safety departments. Table 1 shows respondent characteristics like age, gender and work experience.

**Table 1:** Respondent characteristics (*n* = 211)

Characteristics	Board of directors (*n* = 22)	Chief of medical staff (*n* = 35)	Nursing officer (*n* = 40)	Head of medical department (*n* = 55)	Director of quality and patient safety (*n* = 59)	Overall (*n* = 211)
Age, mean years (SD)	56.1 (5.2)	49.5 (6.3)	46.6 (8.8)	49.7 (6.5)	46.7 (10.8)	48.9 (8.6)
Experience, mean years (SD)^a^	4.6 (3.5)	4.4 (4.8)	3.9 (4.6)	9.2 (4.8)	6.1 (4.8)	6.1 (5.7)
Female, n (%)	5 (22.7)	6 (17.1)	31 (77.5)	11 (20)	37 (62.7)	90 (42.7)

^a^Ten missing cases

### Relative importance of factors

The ranking of the different factors is shown in Table 2. Of the 32 factors, 16 factors had an agreement percentage of at least 90%, two factors had an agreement percentage of 100% and two factors had an agreement percentage less than 50%. Some factors had an agreement percentage that placed them outside the top 10, while their item score and CI were higher than some of the top 10 factors.

**Table 2:** Factors for effective auditing and their ranking

Theme	Factor	Rank order	Agree %	Item score (mean, 95% CI)
Positioning	Audit as an improvement tool as well as a control tool	1	100	5.80 (5.75–5.86)
Culture	Learning culture at department	2	100	5.40 (5.31–5.48)
Organisation	Department is aware of audit purpose	3	99	5.60 (5.52–5.69)
Positioning	Follow-up on auditing results by head of department	4	99	5.40 (5.30–5.49)
Organisation	Audit also focusses on healthcare pathways	5	98	5.37 (5.26–5.47)
Positioning	Audit results are embedded in the planning and control cycle of a hospital	6	98	5.32 (5.21–5.42)
Auditors	Quality of the auditors	7	98	5.17 (5.06–5.29)
Positioning	Dissemination of audit results to all personnel	8	97	5.27 (5.15–5.39)
Organisation	Audit is adjusted fit to individual departments	9	95	5.08 (4.95–5.20)
Culture	Staff feel that audit contributes to patient safety	10	95	4.96 (4.84–5.07)
Auditors	Evaluation of individual auditors	11	95	4.96 (4.83–5.09)
Positioning	Spreading the purpose and value of audit by board of directors	12	93	5.26 (5.12–5.39)
Auditors	Multidisciplinary audit team	13	93	4.99 (4.85–5.14)
Auditors	Auditors coming from other hospitals taking part in the audit team	14	93	4.83 (4.69–4.97)
Culture	Availability of head of department on audit day	15	92	4.89 (4.75–5.02)
Positioning	Board of directors addresses departments when follow-up on auditing result is not going according to plan	16	91	4.92 (4.79–5.06)
Positioning	Departments receive support during improvement actions after audit	17	88	4.72 (4.58–4.86)
Organisation	Auditors get tunnel vision on a department because of selective information during preparations	18	87	4.45 (4.30–4.60)
Organisation	There is room for soft signals, not only facts, in the audit report	19	86	4.71 (4.55–4.87)
Organisation	Patients as an information source in auditing	20	85	4.68 (4.51–4.84)
Culture	Time investment as a barrier	21	82	4.41 (4.24–4.57)
Organisation	Audit also focusses on care provided by healthcare professionals and adverse events	22	81	4.65 (4.43–4.86)
Auditors	Medical specialist on the auditteam	23	79	4.50 (4.32–4.68)
Culture	Staff sees added value of audit	24	78	4.34 (4.18–4.50)
Organisation	Department knows that the audit team is coming	25	75	4.43 (4.24–4.62)
Culture	Quality is ‘part of the job’	26	75	4.35 (4.15–4.54)
Culture	Importance of audit outcomes compared to importance of outcomes of other visitations and instruments	27	66	3.94 (3.78–4.10)
Auditors	Patients as auditors	28	64	3.83 (3.64–4.01)
Organisation	An extensive and detailed audit report	29	59	3.81 (3.62–4.01)
Auditors	Chairman of the audit team is a high profile employee	30	56	3.57 (3.39–3.75)
Auditors	Only employees with a (para)medical education should be auditors	31	37	3.05 (2.84–3.25)
Auditors	The department to be audited influences the composition of the audit team	32	26	2.75 (2.75–2.91)

Abbreviation: *Positioning* board positioning of audits, *Culture* cultural factors and attitudes towards auditing, *Organisation* organisation and content of audits, *Auditors* competences and composition of audit team

The category that was most common in the top 16 of factors was Board positioning of audits (6 factors), followed by Competences and composition of audit team (4 factors), Culture and attitudes towards auditing (3 factors) and Organisation and content of audits (3 factors). The three factors that were ranked as 1 through 3 are related to the purpose of the audit and the culture at the department level. Factors that were ranked as 4 and 6 are related to the follow-up of the audit. Of the six factors with an agreement percentage less than 70%, four factors are related to the theme ‘auditors’. These factors are related to the composition of the audit team, while the factors regarding auditors that have a higher agreement percentage (‘evaluation of individual auditors and ‘quality of the auditor’) are related to the competences of auditors.

### Differences in perceptions between stakeholders

The percentage of agreement per stakeholder group is shown in Table 3. A total of 18 factors had a significant difference in agreement percentage between stakeholders (*P* < 0.05). Of these factors, 14 had a significant difference of at least 20%, which we will discuss below.

**Table 3:** Importance of factors amongst subgroups

Factor	Board of directors (*n* = 22)	Chief of medical staff (*n* = 35)	Nursing officer (*n* = 40)	Medical department head (*n* = 55)	Director of quality and patient safety (*n* = 59)	Overall (*n* = 211)
Category: board positioning of audits						
Audit as an improvement tool as well as a control tool	100	100	100	100	100	100
Spreading the purpose and value of audit by board of directors	94	91	95	87	94	93
Departments receive support during improvement actions after audit	67*^R^	91*	92*	100	78	88
Audit results are embedded in the planning and control cycle of the hospital	94	100	97	98	97	98
Board of directors addresses departments when follow-up on auditing results is not going according to plan	94	87	97	87	90	91
Dissemination of audit results to all personnel	94	100	97	98	95	97
Follow-up on auditing results by head of department	100	100	97	100	98	99
Category: organisation and content of audits						
Department is aware of audit purpose	100	100	97	98	100	99
Audit is adjusted fit to individual departments	78*^R^	97	92	100	97*	95
Department knows that the audit team is coming	67	77	59*^R^	81*	80*	75
Extensive and detailed audit report	44	53	72	57	NA	59
Audit also focuses on health care pathways	100	97	97	94	100	98
There is room soft signals, not only facts, in the audit report	83	97*	90	89	75*^R^	86
Audit also focuses on care provided by healthcare professionals and adverse events	78	82	83	97	NA	81
Patients as an information source during audits	94	89	95*	72^*R*^	86	85
Auditors get a tunnel vision on department because of selective information during preparations	69*^R^	94*	92*	84	88	87
Category: competences and composition of audit team						
Patients as auditors	67	74	78*	57	55*^R^	64
Patients are not suited to be an auditor because they are not objective	22*^R^	29	33	50*	45	39
Patients are not suited to be an auditor because they do not have sufficient knowledge regarding healthcare guidelines	17*^R^	41	41	47*	43	41
Patients are not suited to be an auditor because confidentiality cannot be secured.	17*^R^	41	33	44	51*	41
Quality of the auditors	94	100	97	94	100	98
Evaluation of individual auditors	83*^R^	94	90	98	98*	95
Multidisciplinary audit team	94	94	100	94	86	93
Medical specialist on the audit team	78	88*	79	92*	61*^R^	79
Chairman of the audit team is a high-profile employee	50	68	53	59	51	56
The department to be audited influences the composition of the audit team	17*	29	13*	48*^R^	15*	26
Auditors coming from other hospitals taking part in the audit team	94	97	95	90	91	93
Only employees with a (para)medical education should be auditors	39	30*	46	56* ^*R*^	19*	37
Category: cultural factors and attitudes towards auditing						
Staff sees added value of audit	89	91*^R^	69^*^	82	67*	78
Presence of head of department on audit day	94	94	80*	96*^R^	93	92
Time investment	72	83	72*	90*^R^	83	82
Staff feel that audit contributes to patient safety	94	97	90	96	97	95
Quality is ‘part of the job’	67*	74	71*	90*^R^	66*	75
Importance of audit outcomes	78	62	70	56	69	66
Learning culture at department	100	100	100	100	100	100

*Subgroups differ significantly (*p* < 0.05) with the reference group (^*R*^)

Amongst the boards of directors, a significantly smaller group agreed that departments should get support during improvement actions after audits (67%) than amongst the chiefs of medical staff (91%) and nursing officers (92%) (*P* < 0.05). This trend is also visible when comparing the boards of directors to heads of medical departments (100%).

Of the nursing officers, 59% agreed that it is important that departments know that the audit team is coming. This is significantly lower than the agreement percentage amongst heads of medical departments and directors of quality and patient safety departments (respectively 81% and 80%) (*P* < 0.05). The percentage of directors of quality and patient safety departments who agreed that the inclusion of soft signals (e.g. problems in functioning of individual health care professionals or communication problems in teams) in the audit report contributes to effective auditing (75%) is significantly lower than the agreement percentage amongst chiefs of medical staff (97%) (*P* < 0.05). Almost every nursing officer believed that patients should be an information source during auditing (95%), while a significantly lower percentage of heads of medical departments (72%) shared this opinion (*P* < 0.05). Amongst boards of directors, 69% believed that auditors get tunnel vision on a department because of selective information during preparations, which is significantly lower than the percentages amongst chiefs of medical staff and nursing officers (94% and 92%) (*P* < 0.05).

The percentage of directors of quality and patient safety departments who agreed to the importance of patients as auditors (55%) differed significantly from the agreement percentage (78%) amongst nursing officers. Amongst boards of directors, 22% felt that lack of objectivity would be a barrier for including patients in an audit team, which is significantly lower than the percentage of heads of medical departments who agreed (50%) (*P* < 0.05). The agreement percentage amongst boards of directors (17%) to the fact that lack of knowledge would be a barrier for including patients in an audit team is significantly lower than the agreement percentage amongst heads of medical departments (47%) (*P* < 0.05). Not being able to secure confidentiality would be a barrier for 17% of the boards of directors, while 51% of the directors of quality and patient safety departments agreed, which is significantly higher (*P* < 0.05).

Heads of medical departments (92%) and nursing officers (88%) felt that a medical specialist on the audit team is a precondition for effective auditing. This is significantly higher than the percentage of directors of quality and patient safety departments who agreed (61%) (*P* < 0.05). The same trend is visible when looking at the agreement percentages regarding an audit team consisting of only employees with a (para)medical education. The majority of heads of medical departments (56%) agreed that this influences effective auditing, while a minority of directors of quality and patient safety departments agreed (19%) (*P* < 0.05). Amongst heads of medical departments, 48% agreed to the fact that the department to be audited should have an influence on the composition of the audit team. Amongst the boards of directors, nursing officers and directors of quality and patient safety departments, this percentage is significantly lower (17%, 13% and 15%, respectively) (*P* < 0.05).

Amongst chiefs of medical staff, 91% agreed that staff seeing the added value of audits is necessary for effective auditing, which is significantly higher than the agreement percentage amongst nursing officers (69%) and directors of quality and patient safety departments (67%). Heads of medical departments (90%) regarded quality being part of the job as an important facilitator for effective auditing, with an agreement percentage that was significantly higher than the agreement percentage amongst boards of directors (67%), nursing officers (71%) and directors of quality and patient safety departments (66%) (*P* < 0.05).

## Discussion

This study investigated the importance of factors that hinder or stimulate the effective use of audits by hospital boards and executives to govern patient safety. Our findings indicate that almost all factors found in the interviews with persons involved with governance and audits in a previous study [13] matter when trying to improve audits. First, effective auditing depends on the extent to which boards successfully demonstrate the value of the audit and are able to follow up on the auditing results. We found this in earlier studies on auditing as well [13, 14, 27]. Leadership through agenda setting and creating a sense of urgency is already described in the literature as an important influence on the effectiveness of quality-management systems and clinical governance [8, 28–31]. Our study underscores the importance of leadership in auditing, while this is often missing in practice [13]. Second, the quality of auditors and structural evaluation of their competences influences effective auditing, as is in line with literature on auditing [13, 16]. However, structural evaluation of the competences of auditors is not common in practice [13]. Third, we found that audits can be more effective when adjusting their content to fit auditees’ needs, as well as making sure that auditees know why the audit team is coming [13]. Finally, this study has shown that a learning culture, in which staff are eager to learn from safety problems and are willing to improve their work [13], is indeed an important influence on auditing. Low-scoring factors, namely ‘only employees with a (para)medical education should be auditors’ and ‘the department to be audited influences the composition of the audit team’ seem to revolve around the composition of the audit team. This suggests that respondents find the quality of individual auditors more important than the background of auditors.

Investigating the differences between stakeholders helped us understand some of these outcomes. In the example mentioned above, we found that medical department heads did agree to these factors, in contrast to other stakeholders. We believe that an explanation for this is the difference between feasibility and desirability. For medical specialists, it is important to talk to their peers during audits; they find it important that the auditor has experience with their type of work, and they value talking to their ‘equal’ [13, 32]. Directors of quality and patient safety departments, however, may know from experience that it is not easy to include medical specialists in the audit team, so if that is a precondition for an audit, getting an audit team together might not even be possible [13]. Another example of the gap between desirable and feasible is patient involvement in auditing. Patients are becoming more and more central players in healthcare and they are increasingly involved in policy making [33] and guideline development [34].

Discussion arises, however, whether and how patients should be involved in the audit process [13]. Our findings show that patient participation in an audit team is not as easy as it seems. Especially medical department heads and directors of quality and patient safety departments seem to lean towards the fact that bias, lack of knowledge and the risk of not securing confidentiality are barriers for involving patient in the audit team. It is curious that the different, more subjective patient experiences are valued less, while confidentiality is not an exclusive characteristic of healthcare professionals.

A final example is the inclusion of soft signals in the audit report, which is very important for chiefs of medical staff and less so for directors of quality and patient safety departments. We suppose that this is because auditors are afraid to lose support amongst auditees when not sticking to the facts, while the chief of medical staff might want to know what exactly is going on between medical specialists [13]. Compared to quantitative monitoring methods, e.g., mortality rates and incident reporting, audits enable the identification of safety problems and their underlying causes because of the qualitative methods, such as interviews and observations. These instruments can provide clearer insight on the actions that must be taken in order to improve patient safety [35]. However, this study shows that some stakeholders raise objections regarding the feasibility of actually including soft signals in the audit report.

A strength of this study is the fact that 91% of all acute-care hospitals in the Netherlands participated in this study, suggesting that the included hospitals and respondents are representative of the Netherlands. We feel that this contributes to a high internal validity of the results. Moreover, the inclusion of a broad range of stakeholders involved in governance and auditing, e.g., hospital boards, clinicians, quality managers, made it possible to investigate in the importance of factors amongst different stakeholder groups.

A limitation is the response rate amongst boards of directors (25%), as this seems like a low rate. However, compared to other literature, this looks like an acceptable rate for this type of respondent. Another limitation is that, since we wanted to present neutral statements in the questionnaire, the difference between a barrier and a facilitator got lost in the questionnaire [36, 37]. However, the absence of certain factors can been seen as a barrier for optimal patient safety auditing and presence of a factor as facilitating.

This study has several implications for practice. Our findings indicate that it is important not just to position the audit as a control tool, but to demonstrate that it can be used as an improvement tool as well. When healthcare professionals perceive an audit as a control tool only, it can be experienced as a tick box-activity [16]. However, when staff experiences that they can learn from auditing to improve their daily practice, they are more likely to involve in auditing [13]. It is important that boards emphasise and explicitly support the value of the audit and state that audits are an instrument to improve patient safety. Boards should communicate to auditees that improving patient safety is at stake, that this is a top priority to them and that the audit is an instrument to facilitate this through monitoring, reflection and learning. Moreover, boards should invest in creating a culture in which auditing is part of the job. In order to do this, boards should engage in active leadership activities, such as agenda setting, to communicate this purpose to auditees [38, 39]. Not only might this positively influence the value of the audit for auditees, but it may also influence the culture at department level [40, 41], creating a favourable culture for auditing. An example of this agenda setting is including audit results in the planning and control cycle and discuss the progress of the improvement actions based on the audit results with the medical head of departments [13]. Finally, an audit is a useful instrument to give insight into potential safety problems originating from more diffuse social and cultural aspects of healthcare (so called ‘soft signals’), e.g., distrust, conflicts, rivalry between staff members, because of its qualitative nature [13]. However, actually including these signals in the audit report does not always seem desirable. We can learn from safety walkarounds, as these enable boards to systematically grasp soft signals through participation [42] without the need for an auditor to include the signals in the audit report.

This study showed that almost every factor we investigated is perceived as important for improving audits. We synthesised these 30 factors into eight practical recommendations for hospitals to implement in practice. These recommendations can be found in Table 4 and can be used as a framework for hospitals to improve their audits.

**Table 4:** Eight recommendation to optimise audits

Eight recommendations to optimise audits	
Equal focus	
Strive for the same goal together. Boards demonstrate and auditees feel that the audit is a chance to improve.	
Learning culture	
Create a culture in which employees are open for feedback and quality is part of the job. Crew Resource Management (CRM) training helps teams to speak up and improves communication within team.	
Audit on board’s agenda	
Include audit results in the planning and control cycle of the hospital. And boards should discuss the progress of planned improvement actions based on the audit results with the head of the departments.	
Post-audit support	
Help health care professionals to improve their health care based on the audit results. Provide support for departments to implement improvements after the audit.	
Quality assurance of auditors	
The quality of an audit depends on the quality of the auditors. Train and evaluate individual competences and skills.	
Peer-to-peer approach	
An audit team should be multidisciplinary. Include nurses and medical specialists in the audit team.	
Soft signals in audit	
Give feedback to departments regarding signals indicating potential safety problems originating from more diffuse social and cultural aspects of healthcare.	
Audit tailoring	
Adjust content of audit to needs and relevance of auditees. Needs should be inventoried before an audit takes place with a self-assessment.	

Certain recommendations (a medical specialist on the audit team, soft signals in the audit report and post-audit support) are based on factors that had a low agreement percentage amongst certain stakeholders, probably relating to these stakeholders’ perceptions of the feasibility of the factors. This is a challenge when implementing this framework. Future research should focus on the role of patients in the audit process. Issues like assuring confidentiality are not restricted to patients, but are issues that arise with every auditor - patient or not. Patient involvement is important in quality measurement [43], and we feel that it is an ethical obligation of hospitals to bring in various backgrounds of knowledge and experience into patient safety programmes, including audits.

## Conclusion

In order to improve hospital-wide audits, hospital boards and executives need to invest in four different areas: the positioning of the audits, their organisation and content, the competences and composition of audit teams and the culture and attitudes concerning auditing. We suggest eight recommendations to optimise audits enabling hospital boards and executives to become more in control and to fulfill their responsibility for patient safety in their hospital.

## Additional file

Additional file 1: Questionnaire (PDF 315 kb)

## Abbreviation

CI: Confidence interval
