# Supplementary material for: Important factors for effective patient safety governance auditing: a questionnaire survey
Source: BMC Health Serv Res. 2018 Oct 20;18:798. doi: 10.1186/s12913-018-3577-9 (PMC6195966; doi:10.1186/s12913-018-3577-9)
Supplement: Supplementary file 1 — Questionnaire (PDF 315 kb) [file 12913_2018_3577_MOESM1_ESM.pdf]

Questionnaire internal auditing

Gender: ☐ male ☐ female

Age: …

Function:
- ☐ Member of the Board of directors
- ☐ Chief of medical staff
- ☐ Nursing officer
- ☐ Head of medical department
- ☐ Director of the quality and patient safety department

Years of experience: …

Name of the hospital: ………………..

Hospital type:
- ☐ Academic medical center
- ☐ Tertiary teaching hospital
- ☐ General hospital

Statements regarding effective internal auditing

	fully disagree	disagree	slightly disagree	slightly degree	agree	fully agree	cannot judge
Important that audit is seen as an improvement tool as well as a control tool	☐	☐	☐	☐	☐	☐	☐
Important to spreading the purpose and value of audit by the board of directors	☐	☐	☐	☐	☐	☐	☐
For commitment for the internal audit it is important that the department is aware of the purpose of the audit	☐	☐	☐	☐	☐	☐	☐
Customisation: it is important to tailor the audit to individual departments	☐	☐	☐	☐	☐	☐	☐
It is important that the department knows that the audit team is coming	☐	☐	☐	☐	☐	☐	☐
An extensive and detailed audit report is more important than a concise audit report	☐	☐	☐	☐	☐	☐	☐
Audit also focuses on health care pathways	☐	☐	☐	☐	☐	☐	☐
Important is that there is room soft signals, not only facts, in the audit report	☐	☐	☐	☐	☐	☐	☐
Audit should also focuses on care provided by healthcare professionals and adverse events and not only on organisational preconditions	☐	☐	☐	☐	☐	☐	☐

	fully disagree	disagree	slightly disagree	slightly degree	agree	fully agree	cannot judge
Patients should be an information source during audits	☐	☐	☐	☐	☐	☐	☐
Patients should act as auditors to involve the patient perspective	☐	☐	☐	☐	☐	☐	☐
Patients are not suited to be an auditor because they are not objective	☐	☐	☐	☐	☐	☐	☐
Patients are not suited to be an auditor because they do not have sufficient knowledge regarding healthcare guidelines	☐	☐	☐	☐	☐	☐	☐
Patients are not suited to be an auditor because confidentiality cannot be secured	☐	☐	☐	☐	☐	☐	☐
Auditors get a tunnel vision on department because of selective information during preparations	☐	☐	☐	☐	☐	☐	☐
The quality of the auditors determine the quality of the audit results	☐	☐	☐	☐	☐	☐	☐
Evaluation of individual auditors is important for the quality of the audit process	☐	☐	☐	☐	☐	☐	☐
A multidisciplinary audit team, including nurses and physiotherapists is a prerequisite	☐	☐	☐	☐	☐	☐	☐
Medical specialist on the audit team is a prerequisite	☐	☐	☐	☐	☐	☐	☐

	fully disagree	disagree	slightly disagree	slightly degree	agree	fully agree	cannot judge
The chairman of the audit team should be a high-profile employee	☐	☐	☐	☐	☐	☐	☐
The department to be audited should have influence on the composition of the audit team	☐	☐	☐	☐	☐	☐	☐
Auditors coming from other hospitals should take part in the audit team	☐	☐	☐	☐	☐	☐	☐
Only employees with a (para)medical education should be auditors	☐	☐	☐	☐	☐	☐	☐
It is necessary that departments receive support with the implementation of improvement actions after internal audits	☐	☐	☐	☐	☐	☐	☐
It is necessary that audit results are embedded in the planning and control cycle of the hospital for effective internal audits	☐	☐	☐	☐	☐	☐	☐
It is necessary that board of directors addresses departments when follow-up on auditing results is not going according to plan	☐	☐	☐	☐	☐	☐	☐
It is important to disseminate the audit results to all personnel on the department	☐	☐	☐	☐	☐	☐	☐
Staff sees added value of audit	☐	☐	☐	☐	☐	☐	☐
The presence of the head of department on the audit day is important	☐	☐	☐	☐	☐	☐	☐

	fully disagree	disagree	slightly disagree	slightly degree	agree	fully agree	cannot judge
The time investment is a limiting factor for the commitment for the internal audit	☐	☐	☐	☐	☐	☐	☐
Staff should feel that audit contributes to patient safety is important	☐	☐	☐	☐	☐	☐	☐
Internal auditing is useful when staff sees quality as 'part of the job'	☐	☐	☐	☐	☐	☐	☐
Audit outcomes are of great importance, despite the amount of other quality measurements	☐	☐	☐	☐	☐	☐	☐
A learning culture at department is important for the effectiveness of internal auditing	☐	☐	☐	☐	☐	☐	☐

Do you have any comments? Please write them here:

....
